# A Message from the Human Placenta: Structural and Immunomodulatory Defense against SARS-CoV-2

**DOI:** 10.3390/cells9081777

**Published:** 2020-07-25

**Authors:** Nina-Naomi Kreis, Andreas Ritter, Frank Louwen, Juping Yuan

**Affiliations:** Division of Obstetrics and Prenatal Medicine, Department of Gynecology and Obstetrics, University Hospital Frankfurt, J. W. Goethe-University, Theodor-Stern-Kai 7, D-60590 Frankfurt, Germany; Andreas.Ritter@kgu.de (A.R.); louwen@em.uni-frankfurt.de (F.L.)

**Keywords:** SARS-CoV-2, COVID-19, placenta, vertical transmission, immune defense, miRNA, interferon type III, NF-κB pathway

## Abstract

The outbreak of the coronavirus disease 2019 (COVID-19) pandemic has caused a global public health crisis. Viral infections may predispose pregnant women to a higher rate of pregnancy complications, including preterm births, miscarriage and stillbirth. Despite reports of neonatal COVID-19, definitive proof of vertical transmission is still lacking. In this review, we summarize studies regarding the potential evidence for transplacental transmission of severe acute respiratory syndrome coronavirus 2 (SARS-CoV-2), characterize the expression of its receptors and proteases, describe the placental pathology and analyze virus-host interactions at the maternal-fetal interface. We focus on the syncytium, the barrier between mother and fetus, and describe in detail its physical and structural defense against viral infections. We further discuss the potential molecular mechanisms, whereby the placenta serves as a defense front against pathogens by regulating the interferon type III signaling, microRNA-triggered autophagy and the nuclear factor-κB pathway. Based on these data, we conclude that vertical transmission may occur but rare, ascribed to the potent physical barrier, the fine-regulated placental immune defense and modulation strategies. Particularly, immunomodulatory mechanisms employed by the placenta may mitigate violent immune response, maybe soften cytokine storm tightly associated with severely ill COVID-19 patients, possibly minimizing cell and tissue damages, and potentially reducing SARS-CoV-2 transmission.

## 1. Introduction

The coronavirus disease 2019 (COVID-19), caused by severe acute respiratory syndrome coronavirus 2 (SARS-CoV-2), has been declared as a pandemic by the World Health Organization (WHO) on March 11, 2020 [1]. As of July 02, 2020, there have been 10,533,779 confirmed cases of COVID-19, including 512,842 deaths (WHO dashboard) [2]. The pandemic poses a global public health crisis, which could also affect next generations.

Questions relating to the safety of pregnancy and the unborn child have arisen. In general, viral infections may predispose pregnancy towards preterm birth, which may have major long-term health implications for newborns [3]. Certain viral infections during pregnancy can lead to adverse pregnancy outcomes, like spontaneous abortion, mother-to-child transmission (vertical transmission) resulting in congenital viral syndromes, fetal growth restriction or stillbirth [4]. Overall, data from several maternal viral pneumonias, including influenza, varicella, rubella [5,6], SARS-CoV [7] and Middle East respiratory syndrome coronavirus (MERS-CoV) [8], suggest that pregnant women are more susceptible to SARS-CoV-2 infections resulting in adverse outcomes. The severity of the disease is associated with overweight and obesity, as well as diabetes, gestational hypertension, or preeclampsia (PE) [9,10,11,12].

Based on data from recent studies dealing with potential SARS-CoV-2 entry mechanisms into the human placenta, the possibility of vertical transmission and placental pathology upon infection, we describe the placenta as a potent physical and immunological barrier employing its multiple cellular and molecular defense pathways against SARS-CoV-2 infection during pregnancy.

## 2. The Placenta in Virus Defense

### 2.1. A Structural and Physical Barrier for SARS-CoV-2

The placenta, containing floating and anchoring villi, is a transient organ made of maternal and fetal tissues [13,14] that harbors two main responsibilities: to nourish and to protect the fetus [15]. Villi are covered by a non-proliferative and multinucleated syncytiotrophoblast (STB or syncytium) [13,14], which is formed and maintained by fusion of an inner layer of proliferative progenitor cells, called villous cytotrophoblasts (vCTBs) [13,14]. During pregnancy, vCTBs of the anchoring villi are able to grow out into the maternal decidua [16]. In these proliferative cell columns, the vCTBs differentiate into an invasive phenotype, termed extravillous trophoblasts (EVTs) [16]. EVTs invade into the maternal decidua, consisting of approximately 40% immune cells [17], where they differentiate into multinuclear giant cells in the myometrium or colonize the lumen of spiral arteries, which are remodeled for the sufficient blood supply and transfer of nutrients to the embryo [16]. The core of the chorionic villi contains several cell types, including immune cells, like Hofbauer cells (fetal macrophages) that are located adjacent to fetal capillaries, fibroblasts, fetal endothelial cells and mesenchymal stem/stromal cells (MSCs) [18,19]. On both villi types, the STB forms the outermost cell layer and is, thus, the key interface between maternal and fetal blood [20], responsible for nutrient exchange and hormone production, including human chorionic gonadotropin and progesterone supporting pregnancy [21].

The intrinsic defense of the placenta is rooted in its architecture: first, the outermost STB layer is periodically regenerated [9,10] and covered by a dense network of branched microvilli that spans an area of 12–14 m^2^ at the end of human gestation [13,22]. Importantly, the fused multinucleated STB layer does not contain intercellular gap junctions [23] that can be exploited by pathogens or modulated by inflammatory signals.

Second, the STB contains an unusually dense cytoskeletal network creating a shielding brush border at the apical surface [24]. Interestingly, based on a murine trophoblast stem cell model, it has been shown that the disruption of actin polymerization with cytochalasin D decreases syncytial elasticity and correlates with increased bacterial invasion, suggesting an important involvement of the actin cytoskeleton in host defense mechanisms [25].

Third, different cellular receptors are used for pathogen recognition or entry [19]. The STB barely expresses toll-like receptors (TLR) or internalization receptors as E-Cadherin, which could recognize pathogens or mediate their cell entry [15,26]. TLRs are key components of the innate immune system that recognize conserved sequences on the surfaces of pathogens initiating effector cell functions [26]. Intriguingly, the expression of TLRs is not continuous throughout pregnancy. In first trimester placenta, EVTs and vCTBs express TLR-2 and TLR-4, while the STB layer is negative for both receptors [26]. By contrast, in term placenta, the expression of TLR-2 and TLR-4 is restricted to the STB and EVTs [27]. More importantly, the STB expresses hardly E-cadherin and is therefore highly resistant to *Listeria monocytogenes* infections, whereas EVTs abundantly express E-cadherin and serve as primary entry portal from both intracellular and extracellular compartments [23].

Fourth, the STB does not express caveolins [28,29], which play a role in intracellular and intercellular signal transduction [30]. Their role in endocytosis and transcytosis allows some viruses to enter host cells [29]. While caveolins are expressed in placental endothelium, stroma cells, smooth muscle cells as well as pericytes [29,31], they are however not [28,29] or only weakly expressed in term STB [31]. This may implicate a physiological defense mechanism developed by the STB against virus-mediated cell damage and vertical transmission, as suggested by Celik and colleagues [32]. As the coronavirus family does not use caveolins to enter cells, the authors hypothesize that SARS-CoV-2 may cluster on caveolins to allow their colonization outside of cells triggering local inflammation and damage [32]. In fact, caveolin-1 (Cav-1) initiates inflammation through the nuclear factor kappa-light-chain-enhancer of activated B cells (NF-κB) pathway triggering the increased release of cytokines like interleukin 6 (IL-6) and tumor necrosis factor α (TNFα) [33]. Due to the lack of sufficient Cav-1 expression, the STB could be protected from virus-related cell damage [32].

Fifth, the basement membrane lying below the vCTB layer represents an additional physical barrier [34]. Taken together, the human placenta, especially the STB, provides a potent structural and physical defense barrier against the majority of pathogens. Immunological defense mechanisms will be discussed below (Section 4).

### 2.2. Pathogens May Pass through the Placental Barrier

Being constantly subjected to maternal blood, the STB is not always capable of preventing all pathogens from damaging and crossing the placental barrier. This barrier may also not work well in early pregnancy stages where intercellular fusion is not fully completed, or in late pregnancy stages where syncytium formation starts to decline [15,35]. Additionally, immunocompromising pathogens can disrupt the physiological defense of the placenta and allow mother-to-child transmission [32]. Maternal disorders, like PE, which suppress the intercellular fusion and syncytium formation [36], can leave the fetus prone to viral infections [32]. Similarly, a high virus load or simultaneous attacking of pathogens may lead to the breakdown of the STB defense [15].

If pathogens break through this barrier, viral infections can result in detrimental defects, including miscarriage, stillbirth, fetal sepsis, premature delivery, fetal growth restriction, birth defects as microcephaly or congenital heart disease, as well as perinatal mortality [4]. Some of the most common pathogens that are able to cross and infect the placental barrier are referred to as TORCH (***T****oxoplasma gondii*, **o**thers (including varicella zoster virus, parvovirus B19, human immunodeficiency virus (HIV), enteroviruses, *Listeria monocytogenes* and *Treponema pallidum* causing syphilis), **r**ubella, **c**ytomegalovirus (CMV), and **h**erpes simplex virus (HSV)) [37,38]. Additionally, the Zika virus (ZIKV) has emerged as the newest TORCH member [37].

For infections, pathogens employ varied strategies to bypass the defense mechanisms and enter a host cell. The general steps of virus infection include host receptor/co-receptor recognition, uptake into the host cell, uncoating of the viral genome and hijacking cellular processes for viral replication [39]. For this, the virus enters the host cell by two main mechanisms: through direct receptor-mediated fusion with the cell membrane (enveloped viruses) or through clathrin-mediated endocytosis [40,41]. In addition, there are also clathrin-independent endocytotic pathways, including caveolin-dependent endocytosis, macropinocytosis, or poorly characterized uptake mechanisms involving neither clathrin nor caveolin like lipid rafts-mediated endocytosis [42,43].

Viruses often target multiple cell types and their transmission can also occur via cell-to-cell spread, which may not depend on cellular receptors and effectively contributes to viral pathogenesis [39]. Several placental cell types can be used as replication sites of pathogens including EVTs, maternal immune cells of the decidua, trophoblast giant cells, Hofbauer cells or vCTBs [19,20]. For instance, to reach vCTBs, human cytomegalovirus (HCMV) bypass the STB layer by receptor-mediated transcytosis [20] using the neonatal Fc receptor (FcRn), which transports immunoglobulin G (IgG) in the second half of pregnancy [44]. Pathogens may further infect Hofbauer cells, which can contribute to vertical transmission as they can serve as viral reservoirs [45]. ZIKV or HIV can conquer the physical barrier through infected maternal blood macrophages (PBMC), which transmit the infection to placental trophoblasts [19]. In the case of SARS-CoV-2, if it is able to enter PBMCs, it does not seem to be replicative in these cells, since blood samples from COVID-19 patients barely displayed viral reads in the transcriptome sequencing of PBMCs [46]. However, as apoptosis of the STB increases, its integrity decreases throughout pregnancy [47,48]. Small injuries containing fibrinoid that are caused by STB shedding, shear or hypoxic stress, and immune-mediated injury from maternal antibodies or the complement system, may facilitate vertical transmission, especially during later gestation [35].

## 3. SARS-CoV-2 and Pregnancy

### 3.1. The Placenta: Receptors and Proteases for SARS-CoV-2 Entry

Coronaviruses, enveloped viruses with a single-strand, positive-sense RNA, enter the target cell by receptor binding, often accomplished by acid-dependent proteolytic cleavage of the spike (S) protein followed by endocytosis, genome replication and exocytosis of mature virions [49]. The S protein of SARS-CoV-2 is the key for the entry into host cells, it mediates receptor recognition and facilitates fusion with the cell membrane [50]. Recent studies have shown that the human angiotensin-converting enzyme II (ACE2) is the host receptor for SARS-CoV-2 [51]. Moreover, based on bioinformatics approaches and protein docking models, it has also been proposed that SARS-CoV-2 binds to human dipeptidyl peptidase 4 (DPP4) with a high affinity [52,53]. As a third alternative receptor, SARS-CoV-2 may attach to cluster of differentiation 147 (CD147)/Basigin (BSG), which facilitates viral invasion [54]. To be fully functional, the S protein of SARS-CoV-2 has to be proteolytically cleaved by human transmembrane protease serine 2 (TMPRSS2) [55], cathepsin L (CTSL) [55], furin, elastase, factor X or trypsin [56,57].

For SARS-CoV-2 to be able to infect the placenta, cells must harbor the abovementioned receptors and proteases. Multiple studies using immunohistochemistry (IHC) or transcriptomic re-analysis of public datasets (microarray or single-cell RNA-sequencing (scRNA-Seq) technology) have investigated the expression of the receptors and priming proteases in various cell types of the maternal-fetal interface (Table 1). 

Valdes and colleagues reported the localization of the ACE2 receptor on the STB, vCTBs, the endothelium, vascular smooth muscle cells, EVTs and decidual cells [58]. In particular, ACE2 mRNA and protein were reported to be highly abundant in early gestational placenta, especially in the STB and villous stroma, and in a lesser extent in vCTBs [59]. Intriguingly, the abundancy of ACE2 and TMPRSS2 varies during pregnancy. Based on scRNA-seq data [61], Li and colleagues revealed that ACE2 is widely spread in specific cell types of the maternal-fetal interface, including stromal cells and perivascular cells of the decidua, and vCTBs and the STB [60]. In contrast, ACE2 is not expressed in EVTs in early gestational placenta (6–14 gestational weeks). The protease TMPRSS2 is abundant in vCTBs and epithelial glandular cells, but low in the STB [60]. The reanalyzing of another single cell transcriptomic study [62] confirmed the mRNA expression of ACE2 and TMPRSS2 in vCTBs and the STB [60]. In addition, ACE2 and TMPRSS2 are low in EVTs at early placenta (eight weeks) and they are significantly increased in EVTs at later gestation (24 weeks), both are co-expressed in vCTBs, the STB and EVTs [60]. Ashray et al. showed the co-expression of ACE2 and TMPRSS2 in 14% of first trimester STB and 15% of second trimester EVTs [65]. Together, these data suggest that ACE2 and TMPRSS2 are co-expressed in vCTBs, the STB and EVTs in the maternal-fetal interface. Interestingly, others revealed that ACE2 and TMPRSS2 are co-expressed in a portion of human epiblast cells, suggesting that early human embryos could be susceptible to infection by SARS-CoV-2 [67]. In rat, ACE2 is enriched in the primary and secondary decidual zone, and in luminal and glandular epithelial cells in early gestation, while its staining is visualized in the labyrinth placenta, and amniotic and yolk sac epithelium in late gestation [78,79]. Moreover, the abundancy of ACE2 in rat placenta increased from mid-gestation [80].

However, further observations are not in line with this assumption. Zheng et al. reported that ACE2 mRNA is low in all maternal-fetal interface cells that are derived from first trimester decidua and placenta, although it is relatively high in decidual perivascular cells [70]. While ACE2 mRNA is still detectable in the STB, vCTBs, decidual perivascular and stromal cells, TMPRSS2 mRNA is below a detectable level [71]. Only a few placental cells and chorioamniotic membranes co-express ACE2 and TMPRSS2 throughout gestation [72]. Low levels of ACE2 and TMPRSS2 were also reported by another study [76]. These data support the notion that the relative absence of one or both ACE2 and TMPRSS2 would be less likely to cause transplacental infection, as the co-presence of both is crucial for SARS-CoV-2 entry into cells. The conflicting scRNA-seq data analyses concerning ACE2 and TMPRSS2 expression levels may arise from varied samples that are derived from variable placental regions and at different gestational ages, various cell isolation/sorting methods and diverse techniques evaluating mRNA or data processing including quality control. Further investigations are required to obtain more homogenous results regarding when and which cell types express the receptors and proteases for SARS-CoV-2 entry at the maternal-fetal interface.

Furthermore, other receptors and proteases mediating SARS-CoV-2 entry into cells are also enriched in the placenta. Recently, datasets from scRNA-seq revealed that DPP4 (CD26) is highly expressed throughout gestation, especially in the STB, vCTBs and EVTs [76], and also in in all stages of human embryonic development [67]. Moreover, DPP4 has been shown to be expressed on placental EVTs suppressing their invasion [81]. Also, CD147, the third suggested entry receptor for SARS-CoV-2 [54], plays various important physiological roles in reproductive tissues including the placenta [82]. CD147 is required for normal implantation by mediating gene expression in mouse uterine stromal cells during pregnancy [83]. It is also expressed in trophoblast cells of human, mouse and rat placenta [84,85,86]. Functionally, CD147 is involved in trophoblast-endometrial cell interaction, trophoblast cell invasion and syncytialization [87]. CD147 has also been proposed to be expressed in MSCs of human cord blood and bone marrow origin [88], indicating that placental MSCs may also express this receptor, making these cells vulnerable to SARS-CoV-2 infection. Most importantly, CD147 as well as the activating protease CTSL, are both expressed during human embryonic development, as revealed from scRNA-seq datasets of human embryos [67]. Both are abundant in almost all placental cells of the first trimester, including the STB, vCTBs, EVTs and villous stromal cells, as well as in EVTs that are derived from the second trimester [65]. Pique-Regi and colleagues found that the placenta and chorioamniotic membranes express high levels of CD147 throughout pregnancy [72]. Interestingly, CD147 levels were high in the blood of preeclamptic women [89]. This may deteriorate the systemic condition of pregnant women with COVID-19 and render the placenta more susceptible to SARS-CoV-2 infection. In addition, the protease CTSL is highly expressed in the STB, vCTBs and EVTs throughout gestation [76], whereas furin is expressed in the STB and involved in syncytialization [90,91]. Furin is also found to be highly expressed in placental villi of both rhesus monkeys and humans during early pregnancy promoting trophoblast cell migration and invasion [92]. Of note, furin as well as CTSL are highly expressed in human placental tissues throughout gestation [72], while furin is especially expressed in ACE2+/TMPRSS2+ STB and EVTs [65].

Taken together, the cellular and molecular composition of the placenta determines the likelihood of SARS-CoV-2 infection and vertical transmission. Although the expression level and period of ACE2 and TMPRSS2 in the placenta require further investigation, other mediators potentially interacting with SARS-CoV-2 are highly abundant. Consequently, they may represent an alternate route for viral infection and eventual vertical transmission.

### 3.2. Potential Evidence for Vertical Transmission of SARS-CoV-2

Vertical transmission is one of the major complications of viral diseases [93]. Regarding SARS-CoV-2, quite limited positive cases were reported, often in the late pregnancy stage with possible postpartum infections. Although studies are emerging, most of them are case reports or small case series. The presence of SARS-CoV-2 has to be confirmed in placental sections, amniotic fluid or cord blood in order to investigate whether the placenta is infected. Interestingly, using transmission electron microscopy (TEM) single SARS-CoV-2 virions were detected in the STB and villous fibroblasts of a woman with severe COVID-19 [94]. Others found SARS-CoV-2 RNA in the placenta and umbilical cord of a symptomatic woman complicated by severe PE [95]. Based on IHC and in situ hybridization (ISH), SARS-CoV-2 spike protein was predominantly observed in the STB. A low amount of virus particles was confirmed within the cytosol of vCTBs, the STB and fibroblasts via TEM [95]. Massive infection with SARS-CoV-2 was also detected in the STB of one placenta of an asymptomatic woman with obesity and a medical history similar to COVID-19 related symptoms but without evidence of vertical transmission indicating that the placenta prevents the passage of SARS-CoV-2 to the fetus [96]. ISH was also used by Patané et al., which showed positive dots for SARS-CoV-2 spike protein mRNA in the STB of two placentas of symptomatic women [97]. RT-PCR positive tested placenta, umbilical blood, amniotic fluid or placental membranes were also observed in rare cases [98,99,100,101,102,103], and viral load was detected at the maternal and fetal side of the placenta [96]. With one exception [103], all women had COVID-19 symptoms [98,99,100,101,102]. In contrast to larger studies showing no evidence of vertical transmission of SARS-CoV-2 [104,105,106], transplacental infection was suspected in three cases of symptomatic women, where neonates had elevated IgM levels suggesting that the neonates were possibly infected in utero, as IgM antibodies are not able to cross the placenta [107,108].

In a recently published systematic review by Walker et al., the risk of neonatal infection by SARS-CoV-2 was estimated to evaluate the likelihood of vertical transmission [109]. They included 49 studies reporting a total of 666 neonates and 28 confirmed COVID-19 infections (4.2%) [109]. The rate of infected neonates was higher with Caesarean section (CS) (5.3%) than with vaginal delivery (2.7%) [109]. According to The International Federation of Gynecology and Obstetrics (FIGO), vaginal delivery is not contra-indicated in COVID-19 patients [110]. In an earlier review summarizing published data of 179 newborns, the infection rate was about 4.47%, with eight cases of potential vertical transmission [111]. Recently, a systemic review of 37 studies, including 275 pregnant women and 248 neonates, was published with quality assessment [112]. The majority of pregnant women had mild to moderate symptoms, two stillbirth and 8% of the neonates were SARS-CoV-2-postive by RT-PCR, but the rate of premature birth was 28% [112], whereas the global preterm rate was reported to be 10.6% [113]. To deliver a full updated picture, we have also summarized published reports with potential evidence of vertical transmission and positive tested neonates, indicating that transmission of SARS-CoV-2 can occur but is still rare (Table 2).

Overall, there is minor evidence for vertical infection, but SARS-CoV-2 virions are able to enter the STB. There are increasing reports of neonatal COVID-19 infections postpartum; however, it is currently elusive whether they are caused by transplacental or horizontal transmission through direct contact shortly after delivery. Definitive proof of vertical infections is still lacking because of limited reported obstetric cases. Well-designed prospective cohort studies with strict inclusion and exclusion criteria are urgently needed to precisely determine the risk of transplacental transmission. It is also necessary to have more definitive evidence as well as “clear definitions” of the term “vertical” transmission [126].

### 3.3. Placental Pathology Caused by SARS-CoV-2

Histopathological examinations of placental tissue from COVID-19 patients can provide valuable insights on fetal progression and neonatal outcome. However, these studies are currently very limited. In the first study with three third trimester placentas, the authors observed increased degrees of fibrin deposition and syncytial knots accompanied by a chorangioma or massive placental infarction [127]. Baergen and Heller investigated twenty term placentas [128], including their former published five cases [129]. The histology of ten placentas was pathologically conspicuous along with maternal vascular malperfusion (MVM), fetal vascular thrombosis, increased intramural fibrin deposition, stromal-vascular karyorrhexis and/or chronic villitis, resulting in placental insufficiency [128,129]. MVM is a placental injury that is related to altered pathologic maternal blood flow resulting in abnormal oxygenation with possibly adverse perinatal outcomes, including fetal demise [130,131]. Clinically, MVM is often associated with hypertensive pregnancy disorders, including severe preterm PE [130,132]. In a study with 16 placentas (15 third trimester and one second trimester), as compared to controls, the placentas had higher rates of MVM features especially decidual arteriopathy including atherosis, fibrinoid necrosis and mural hypertrophy, a significant increase in intervillous thrombi and elevated incidence of chorangiosis without a significant increase of inflammation [133]. In another study, the two analyzed term placentas showed chronic intervillositis with the presence of CD68-positive macrophage infiltration [97]. Intervillositis and infarction accompanied by inflammatory infiltrate consisted of CD68-positive macrophages [123] or diffuse perivillous fibrin deposition [100] was also seen in two other cases. The placenta of a woman with asymptomatic COVID-19 showed a massive infection with generalized inflammation (presence of M2 macrophages, cytotoxic and helper T cells, and activated B-lymphocytes) characterized by histiocytic intervillositis with diffuse perivillous fibrin and necrosis of the STB [96]. Placental sections of a further case report had focal villous edema and decidual vasculopathy [94], a terminology collectively describing pathologic changes that involve the maternal vascular supply to the placenta [131]. Importantly, the adverse long-term effects of women with MVM and/or preterm birth are an increased maternal risk for developing cardiovascular diseases later in life [130,134]. Hypertensive pregnancy complications, like PE, are also associated with enhanced rates of cardiovascular and metabolic diseases in the later life of mother and child [135]. Recently, the possibility of short- or middle-term adverse consequences on placental pathophysiology was hypothesized because of the observed presence of intervillous hemorrhage and moderate fibrin deposition in placental tissue of an asymptomatic woman [103].

While no severe pregnancy outcomes were observed in women during late pregnancy, there are still rare case reports about intrauterine fetal demise (16th week of gestation) with villous edema and a retroplacental hematoma accompanied by preterm rupture of membranes [133] and stillbirth (19th week of gestation) [102] in the second trimester of women with COVID-19. Baud and Greub reported the frequency of inflammatory infiltrates, increased intervillous fibrin deposition and syncytial knots in the placenta of a patient with obesity, SARS-CoV-2 was detected in placental swabs of submembrane and cotyledon via RT-PCR [102]. In a third case report of a second trimester pregnancy (22th week of gestation) complicated by severe PE and placental abruption with the termination of pregnancy, the sections showed diffuse perivillous fibrin deposition and an inflammatory invasion of intervillous CD68-positive macrophages and T-lymphocytes (histiocytic intervillositis) [95]. It is increasingly being recognized that chronic intervillositis is associated with placental insufficiency and poor perinatal outcome like intrauterine growth restriction or fetal death, especially in the first trimester [136]. Interestingly, the placental pathophysiology of SARS-CoV-2 is similar to that of SARS-CoV patients, in a study including seven women in the second as well as third trimester, three placentas had also increased intervillous fibrin deposition with reduced placental perfusion and two showed extensive fetal thrombotic vasculopathy [137].

To sum up, pathological analyses of placental sections from COVID-19 patients often present increased fibrinoid deposition, enhanced inflammation or MVM including thrombi, all indications for a placental injury leading to fetal distress with possible detrimental long-term effects for the newborns. Interestingly, massive macrophage infiltration associated with fibrin deposition has also been observed in the lung tissue of patients with severe COVID-19 [138]. More comprehensive studies are warranted in order to examine placental pathology with obstetric and neonatal outcome, especially during first trimester pregnancy, where the virus may affect placental function and increase the risk of miscarriage.

## 4. General Immune Defense Pathways of the Human Placenta

The placenta is a key immunological barrier against the vertical transmission of pathogens from the mother to the fetus [139,140], which may minimize the possibility of SARS-CoV-2 to colonize the STB barrier and transmit to the unborn child. The maternal-fetal interface, composed of the maternal-derived decidua and the fetal-derived placenta, is potent in immunomodulation. In addition to stromal cells, a remarkably large part (~40%) of the decidua is composed of maternal leukocytes and important for maternal tolerance [140]. In the first trimester decidua basalis, decidual NK (dNK) cells represent the majority of immune cells (~70%), followed by decidual macrophages (20–25%) and T cells, including regulatory T (Treg) cells (3–10%) [17,141]. Maternal leukocytes are recruited by chemokine gradients that are secreted by decidual stromal cells and trophoblasts [142,143], and they typically differ in phenotype and immunomodulatory function from their peripherally circulating counterparts, as excellently reviewed elsewhere [140,144].

In addition to the decidua, the cells of the fetal-derived placenta also contribute to the immunomodulatory defense. The core of the placental villi is enriched with MSCs [13] and they are directly connected to various villous cell types like Hofbauer cells and fetal endothelial cells contributing to villous cellular integrity and homeostasis of fetal blood vasculatures. There is growing evidence that various kinds of placental MSCs have a prominent role in generating a functional microenvironment that is critical for a successful pregnancy [145]. Villous MSCs are potent for differentiation into multiple cell lineages responsible for repair, regeneration, immunomodulation and anti-inflammation reducing tissue damage, like other MSCs do [146]. Hofbauer cells, placental villous macrophages, are present throughout pregnancy [147]. Although, Hofbauer cell populations are antigenically and morphologically heterogeneous, their epigenetic, antigenic and functional profiles are most similar to M2 macrophages [148]. Consistent with an M2-like profile, these cells play an important role in placental development including vasculogenesis/angiogenesis [147] as well as immunomodulation and anti-inflammation [149]. The placenta can also actively transport protective antibodies to the fetus via expression of the IgG receptors neonatal FcRn and FcγRIII on the surface of the STB [150]. This transplacental passage of maternal humoral immunity begins at week 16 of gestation and increases during the course of pregnancy, so that, at term, the fetus has a greater serum concentration of maternally derived IgG than the mother [151].

Collectively, the maternal-placental interface with its unique immune cell distribution provides a modulatory immune defense with a trophoblast-immune cell crosstalk. Importantly, the destruction of the syncytial or decidual barrier triggers a strong innate host defense reaction against pathogens.

### 4.1. The Placenta: Crucial Molecular Signaling Pathways against Viruses

The placenta has evolved several first-line mechanisms to actively protect the fetus against pathogens and prevent vertical transmission: the expression of pattern recognition receptors (PRR) like the well-characterized TLRs by trophoblasts at varied gestational stages (first trimester: vCTBs and EVTs; term: STB and EVTs), amniotic epithelium, decidual and immune cells causing different responses, including caspase activation, cytokine production and inflammatory response [152], or the release of cationic membrane-active antimicrobial proteins and peptides into the amniotic fluid by maternal-fetal membranes (as summarized in [153]). Trophoblasts also express intracellular cytoplasmic-based Nod-like receptors (NLRs) as second-line for pathogen recognition triggering cytokine release [154].

The STB as well as dNK cells, macrophages and lymphocytes located in the maternal-fetal interface form a strong and controlled defense against invading pathogens. Among the aforementioned various regulated molecular immune defense mechanisms, three molecular pathways are especially fundamental: the type III IFN signaling, secreted miRNAs triggering autophagy and the NF-κB pathway.

#### 4.1.1. The Type III IFN Signaling in Immune Defense

One of the major downstream products of the PRRs, including TLR, is the interferon (IFN) family [155]. The antiviral effects of IFNs are against RNA viruses, DNA viruses, intracellular bacteria and parasites [156]. Barrier surfaces, including the human placenta, use IFN production as a potent antiviral response [141]. IFNs initiate a signaling cascade through the Janus kinase signal transducer and activator of transcription (JAK-STAT) pathway, leading to the transcriptional regulation of hundreds of IFN-regulated genes [156]. The more extensively studied type I IFNs control infection systemically, whereas type III IFNs (IFN-λ) regulate it locally at barrier surfaces [157]. The type III IFN family includes IFN-λ1 (IL-29), IFN-λ2 (IL-28A) and IFN-λ3 (IL-29B) [158]. In 2013, IFN-λ4 was discovered inducing antiviral activity against hepatitis C virus in cultured Huh7 liver cells and coronaviruses strain 229E (HCoV-229E) as well as MERS-CoV in human airway epithelial cell culture [159]. Type III IFNs signal through a heterodimeric receptor (IFNLR1/IL10R2) [160]. As part of the innate virus defense, human trophoblasts constitutively release type III IFNs functioning in a paracrine and autocrine manner as known from ZIKV infection [161]. In mice, type III IFNs protect against viral infections, mid-gestation fetuses that lack IFN-λ signaling were more permissive to ZIKV infection, whereas the injection of recombinant IFN-λ2 restricted its vertical transmission [162]. In accordance with this, medium from primary human trophoblast cells isolated from full-term placentas were able to protect non-trophoblast cells from ZIKV infection through the constitutive release of IFN-λ1, which was abolished in cell lines with defective STAT1 pathway [161]. The major cell type responsible for IFN-λ release is the STB, which might deliver IFNs directly into the maternal blood [157]. The inhibition of vCTB fusion reduces the induction of IFN-regulated genes, whereas its stimulation triggers their expression [161]. To sum up, type III IFNs play an important role in the placental antiviral defense, though, the exact molecular targets of this signaling pathway remain to be elusive. Interestingly, given that SARS-CoV-2 infection induces low type I and III IFN levels [163], the potential use of IFNs as a treatment strategy for COVID-19 or synthetic PRR agonists to increase the induction of IFN response are discussed and are currently under investigation [164].

#### 4.1.2. Trophoblastic microRNAs and Autophagy in Immune Defense

A different defense strategy is attributed to the fact that placental trophoblasts produce and release exosomes [165], extracellular membrane vesicles from endocytic origin mediating communication or facilitating antigen presentation [166]. Interestingly, the STB derived from primary human term placenta secrete vesicle-enclosed microRNAs (miRNAs), like chromosome 19 miRNA cluster (C19MC), which restrict viral infections in autocrine and paracrine manners [139,140,167]. Importantly, the antiviral properties of C19MC do not depend on type III IFN signaling [161]. Delorme-Axford et al. observed an inhibition of viral replication and a pronounced up-regulation of autophagy in cells exposed to primary human term trophoblasts conditioned medium conferring this resistance to nonplacental recipient cells, possible due to exosome-packed C19MC miRNAs [139]. Studies with BeWo cells releasing exosome-packed miR517a and miR21 revealed that the STB is the main supplier of released miRNAs [168]. At least three members of the C19MC family (miR517-3p, miR516b-5p, miR512-3p) exhibit these potent antiviral effects against RNA and DNA viruses, and strongly induce autophagy, which is involved in the viral resistance of recipient cells [139]. Autophagy, an evolutionary conserved degradative clearance mechanism, participates in antiviral host defense by targeting cytoplasmic viruses for lysosomal degradation, limiting viral replication and/or interacting with innate immune components, such as TLRs [169,170]. Autophagy is involved in limiting inflammation signals upon virus invasion [171]. In general, basal levels of autophagy are low and stimulated upon cellular stress or virus infection [172] via PRRs [173]. Interestingly, placental trophoblasts, which are highly resistant to virus infection and exhibit high resting levels of autophagy, release exosomes containing C19MC miRNAs [38,139,167]. These exosomes deliver their miRNA cargo to maternal, fetal or placental cells to alter gene expression, culminating in autophagic induction and, subsequently, virus degradation [38,139,167]. Importantly, the inhibition of autophagy with 3-methyladenine, an inhibitor of autophagosome biogenesis, or siRNA-mediated inhibition of beclin-1, a key regulator in the autophagy network [174], abrogated the antiviral effects of C19MC [5,139]. Notably, others demonstrated an involvement of C19MC in cell-to-cell communication between placenta and immune cells, as it is upregulated in third trimester maternal peripheral blood NK cells compared to first trimester [150]. Together, this pathway may constitute a powerful evolutionary adaptation restricting against virus invasion and maintaining trophoblast integrity by transferrable antiviral activity. The use of in vitro-constructed miRNAs as therapeutic target or vaccine against SARS-CoV-2 was recently proposed [175]. The precise mechanisms are unknown and need further investigations.

#### 4.1.3. The Nuclear Factor Kappa B (NF-κB) Pathway in Immune Defense

Upon viral infection of the host cell, the induction of signaling cascades leads to antiviral responses that are mediated by type I IFNs and the NF-κB pathway [176]. NF-κB is a key transcription factor that activates numerous genes that are involved in cellular immune response, inflammation and oxidative stress [177,178]. Of importance, the NF-κB pathway is also implicated in placental development [179]. NF-κB initiates the production and secretion of cytokines, including TNFα, which not only elicits pro-inflammatory cascades, but also serves as a ligand itself to increase NF-κB activity [180,181]. Interestingly, many studies have shown that NF-κB plays an important role in the pathogenesis of lung diseases [182] as well as of pregnancy-related diseases [179]. Most importantly, treatment with drugs that inhibited NF-κB activation led to a reduction in inflammation and lung pathology in SARS-CoV-infected cell culture experiments and mice with a significant increase in the survival rate of mice after SARS-CoV infection [183,184]. Given that inflammation is an important component to normal pregnancies [179], whereas the first and third trimester are described as pro-inflammatory, the second trimester is considered to be anti-inflammatory [185], the regulation of the NF-κB pathway is important for a controlled immune response.

## 5. Perspectives for Future Investigations

We are facing a great body of unanswered questions, especially at the molecular level. Further investigations are required in order to address these questions, for example, how receptors and proteases for the SARS-CoV-2 entry are expressed in each population of the maternal-fetal interface throughout pregnancy, which entry pathways are used by SARS-CoV-2 to infect individual cell populations, how SARS-CoV-2 is finally transmitted to the fetal vessels within villi, how each cell population responds to SARS-CoV-2, and what kind of impact this infection confer on the placental and maternal side. For doing so, various trophoblastic cell lines, placental explant cultures, three-dimensional (3D) organoids and animal models will be helpful in deciphering the roles in the function and alteration of the placenta following SARS-CoV-2 infection. In particular, 3D organoids from first trimester placental trophoblasts [186,187] may open up novel paths for detailed molecular studies regarding SARS-CoV-2 infection and molecular pathways. Moreover, though differences between animal and human placenta, animal models are necessary to understand the dynamic immunological complexities of the maternal-fetal tolerance, the inflammation at the maternal-fetal interface and the disruption of tolerance associated with SARS-CoV-2 infection [140]. Especially, knockout/in mouse models, such as ACE2 knockout mice [188], will be useful to investigate whether ACE2 is the major receptor for SARS-CoV-2 infection in trophoblastic cells.

## 6. Conclusions

Based on the observations from SARS-CoV and MERS infection, and current data from COVID-19 pandemic, it is clear that vertical transmission may occur in rare cases, owing to the fine-regulated immune defense/modulation mechanisms and potent physical barrier in the maternal-fetal interface. Pregnant women with chronic inflammation or vascular defects, including obesity, hypertension or PE may be more susceptible to SARS-CoV-2 infection.

**Figure 1:**
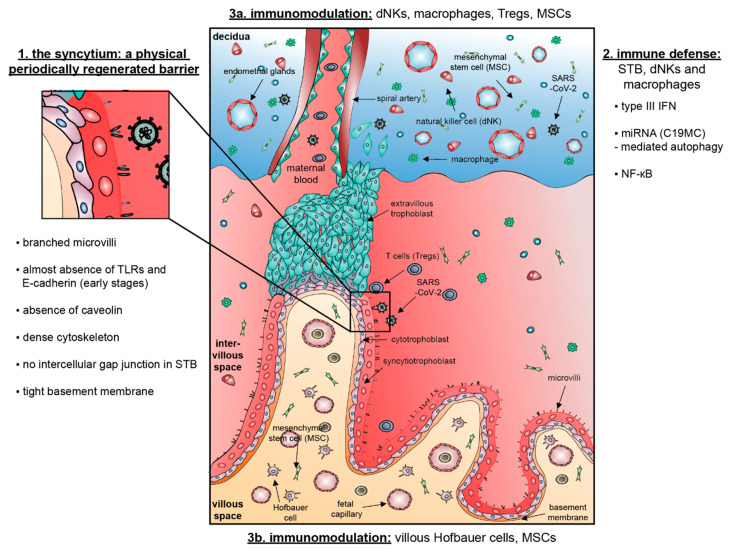
Schematic illustration of the human placenta: physical barrier, immune defense and immunomodulation against SARS-CoV-2.

We assume the following messages from the human placenta: (1) SARS-CoV-2 entry may be physically blocked with all barrier defense mechanisms; (2) SARS-CoV-2 may be actively combated by three molecular pathways (the type III IFN signaling, secreted miRNAs triggering autophagy and the NF-κB pathway); and (3) if infected, immunomodulation could be employed, which may mitigate violent immune response, possibly soften cytokine storm tightly associated with progression of COVID-19, potentially minimizing damages in cells and tissues, and probably reducing SARS-CoV-2 transmission (Figure 1). 

Whether these mechanisms attenuate SARS-CoV-2 transmission remain to be proven. The final outcome after the COVID-19 pandemic will provide a conclusive picture in terms of the frequency and severity of vertical transmission and the impact on both mother and fetus.

## Figures and Tables

**Table 1:** Expression of receptors/proteases for severe acute respiratory syndrome coronavirus 2 (SARS-CoV-2) entry at the maternal-fetal interface and human embryos/fetuses.

Reference	Method/Datasets	Placental Tissue/Patient Information	Receptors/Proteases	Main Results
**Transmission Possible**				
Valdés et al. [58]	IHC	11 early pregnancy failures (5 miscarriages, 9.5 ± 2.2 w; 6 ectopic pregnancies, 7.4 ± 1.9 w), 15 normotensive (38.7 ± 0.9 w) and 10 preeclamptic placentas (35 ± 2.9 w)	ACE2	ACE2 is expressed in placental villi (STB, vCTBs, vascular smooth muscle cells, endothelium) and in the maternal stroma (EVTs, decidual cells). ACE2 expression is comparable between normal term and PE, except for increased ACE2 in umbilical arterial endothelium in PE.
Pringle et al. [59]	mRNA expression IHC	Early gestation placenta (6–16 w, *n* = 14 for IHC, n = 22–26 for mRNA, elective terminations) and term (37-41 w, *n* = 7–9 for mRNA, CS)	ACE2	ACE2 mRNA is highest in early gestational samples with a sharp decline at term. ACE2 protein is abundant in the STB and villous stroma, and in a lesser extent in vCTBs in early gestation placenta; not mentioned for term.
Li et al. [60]	scRNA-seq E-MTAB-6701 [61], GSE89497 [62] and datasets of fetal heart [63], liver or kidney [64]	11 deciduas and 5 placentas from 6–14 w [61]; 1st trimester: villi from 8 w placentas and 2nd trimester: 24 w placenta for EVTs [62]	ACE2 TMPRSS2	ACE2 is highly abundant in decidual stromal cells and perivascular cells, and in vCTBs and the STB. TMPRSS2 is expressed in vCTBs and epithelial glandular cells, and low in the STB. ACE2 expression is significantly increased in EVTs at later stage of pregnancy (24 w). TMPRSS2 has a similar dynamic alteration in the STB. ACE2 and TMPRSS2 are co-expressed in vCTBs, the STB and EVTs. ACE2 is also expressed in specific cell types of human fetal heart and liver.
Ashray et al. [65]	scRNA-seq GSE89497 [62] and [66]	1st trimester: villi from 8 w placentas and 2nd trimester: 24 w placenta for EVTs [62]; 1st trimester (6–11 w) villi (*n* = 8) and decidua (*n* = 6) [66]	ACE2 TMPRSS2 CD147 CTSL FURIN	ACE2 and TMPRSS2 are co-expressed in 14% STB in 1st trimester placenta and in 15% EVTs in 2nd trimester placenta. 18% vCTBs express ACE2. CD147 and CTSL are abundant in almost all EVTs, the STB, vCTBs and villous stromal cells. The ACE2+/TMPRSS2+ STB and EVTs abundantly express FURIN as well as mRNA for proteins involved in viral budding and replication.
Colaco et al. [67]	scRNA-seq of human embryos [68,69]	Human pre-implantation embryos and hESCs [68]; transcriptome signature for human pre-implantation epiblast [69]	ACE2 TMPRSS2 CD147 CTSL DPP4	ACE2 and TMPRSS2 are co-expressed in a proportion of epiblast cells. CD147, CTSL and DPP4 are expressed in all stages of embryonic development. The cells of the epiblast express genes involved in viral endocytosis and replication. Cells of the blastocyst also express DPP4. In ACE2+/TMPRSS2+ cells of the epiblast, biological processes of placental development and viral entry in host cells are enriched.
**Transmission Not Likely**				
Zheng et al. [70]	scRNA-seq E-MTAB-6678 [61]	11 deciduas and 5 placentas from 6–14 w	ACE2	The majority of ACE2-expressing cells are perivascular cells. Its expression is very low in the STB, decidual stromal cells and epithelial glandular cells.
Sungnak et al. [71]	scRNA-seq E-MTAB-6701 [61]	11 deciduas and 5 placentas from 6–14 w	ACE2 TMPRSS2	ACE2 expression is noticeable in certain cell types in the placenta or the decidua (the STB, vCTBs or decidual perivascular cells, decidual stromal cells) *w/o* TMPRSS2.
Pique-Regi et al. [72]	scRNA-seq E-MTAB-6701 [61], NIH dbGAP phs001886.v1.p1 [73], newly generated scRNA-seq/snRNA-seq data [72], microarray datasets E-TABM-577 [74,75]	11 deciduas and 5 placentas from 6-14 w [61]; 3rd trimester placenta: term (38-40 w, *n* = 6) or preterm (33–35 w, *n* = 3), 66.67% CS [73]. Placenta accreta at 18 w and 32 patients at 3rd trimester placenta (32.9–39.1 w, 59.4% CS) [72]. 3rd trimester placenta: spontaneous labor at term w/o (*n*=10) and with villitis of unknown etiology (*n*=10) [74]; PE/SGA/PE+SGA samples (*n* = 28, ~34.2 w) [75].	ACE2 TMPRSS2 CD147 CTSL FURIN	Very few cells co-express ACE2 and TMPRSS2 in any of the three trimesters, also minimally detected in chorioamniotic membranes. Placenta and chorioamniotic membranes express high levels of CD147 throughout pregnancy. CTSL and FURIN are highly abundant in placental tissue throughout gestation. The analyses of bulk gene expression data revealed that ACE2 is detected in most samples, while TMPRSS2 is largely undetected.
Constantino et al. [76]	Affymetrix microarray dataset GSE9984 [77] and scRNA-seq E-MTAB-6701 [61]	Villous trophoblast tissues of 1st trimester (45–59 days, *n* = 4), and 2nd trimester (109–115 days, *n* = 4) from uncomplicated elective terminations and 3rd trimester (*n* = 4) from term, CS [77]; early maternal-fetal interface (11 deciduas and 5 placentas from 6–14 w) [61].	ACE2 TMPRSS2 DPP4 CTSL	Low levels of ACE2 and TMPRSS2, but villous trophoblast cells co-express high levels of DPP4 and CTSL throughout gestation. DPP4 and CTSL are highly co-expressed in the STB, vCTB and EVTs.

ACE2, angiotensin-converting enzyme II; CD147, cluster of differentiation 147; CS, Caesarean section; CTSL, cathepsin L; hESCs, human embryonic stem cells; EVT, extravillous trophoblasts; PE, preeclampsia; scRNA-seq, single-cell RNA-sequencing technology; SGA, small for gestational age; snRNA-seq, single-nuclear RNA-seq; STB, syncytiotrophoblast; TMPRSS2, transmembrane protease serine 2; vCTB, villous cytotrophoblast; w, weeks; *w/o*, without.

**Table 2:** Potential evidence of vertical transmission from pregnant women with COVID-19. 35 cases with SARS-CoV-2 positive newborns (RT-PCR) are identified, whereby we cannot exclude a possible duplication of 13 cases with positive RT-PCR tests reported from Wuhan, China [114,115,116,117,118,119,120].

Reference	Patient Information	Material/Method	Positive Neonates	Possible Vertical Transmission
Algarroba et al. (NY, USA) [94]	28 w + 4 d, 40 y old, CS, severe infection	Placental evaluation using TEM	0	Viral particle detected in the STB and villous fibroblasts.
Alzamora et al. (Lima, Peru) [121]	33 w, 41 y old, diabetes, BMI 35 kg/m^2^, CS, severe infection	RT-PCR	1	Neonatal isolation, positive NP swab 16 h and 48 h postpartum (pp).
Buonsenso et al. (Rome, Italy) [98]	Case 1: 38 w + 3 d, 42 y old, CS, symptomatic COVID-19 with cough Case 2: 35 w+5 d, 38 y old, CS, symptomatic with cough, dyspnea and fever	Serology RT-PCR	1	Case 1: 24 h pp: slightly elevated IgG; 24 h and 3 d pp: negative NP swab; 15 d pp: positive NP swab, in-hospital separation; at home (5 d) breastfeeding with mask; late-onset infection. Case 2: NP swabs negative, RT-PCR positive placenta and umbilical blood; positive breast milk (3 out of 5 samples).
Dong et al. (China) [107]	34 w + 2 d, 29 y, CS, symptomatic	Serology/CLIA	(1) *	Elevated IgM and IgG, IL-6 and IL-10; 2 h pp; elevated IgM antibody level suggests that the neonate was infected in utero. Negative RT-PCR.
Ferraiolo et al. (Genoa, Italy) [103]	38 w + 3 d, 30 y old, CS, asymptomatic woman	RT-PCR	0	Placental swabs obtained from the fetal surface proximate to the umbilical cord were positive for SARS-CoV-2 RNA.
Ferrazzi et al. (Italy) [122]	42 cases, 30 term, mean age 32.9 y old (range 21–44 y), 18 CS/24 VD, 6 with gestational diabetes, generally mild to moderate symptoms	RT-PCR	3	2 newborns had positive NP swabs on day 1 and 3, breastfeeding, skin-to-skin contact allowed. 1 newborn (VD) had a positive test with respiratory symptoms on day 3, without breastfeeding.
Hosier et al. (Connecticut, USA) [95]	22 w, 35 y old, severe PE, elevated transaminases, and low platelets, placental abruption with termination of pregnancy, symptomatic	RT-PCR, sequencing of SARS-CoV-2 RNA (placenta) IHC, ISH and TEM (placenta)	0	SARS-CoV-2-RNA positive placenta and umbilical cord; fetal lungs, heart and kidney were negative. IHC for SARS-CoV-2 spike protein: predominantly in the STB, confirmed with ISH. TEM: virus particles within the cytosol of placental cells (vCTB, STB, fibroblasts).
Hu. et al. (China) [114]	7 cases, 38 w + 2 d to 41 w + 2 d, 30–34 y old, 6 CS/1 VD	RT-PCR	1	1 NP swab positive 36 h postnatal (CS), neonatal isolation for 14 d, formula-fed, symptomatic mother.
Khan et al. (China) [115]	17 cases, mean 38.1 w (35 w + 5 d-41 w), mean 29.29 y old (24—34 y), CS	RT-PCR	2	2 neonates with positive NP swab 24 h postnatal, with 1 developing pneumonia; 4 neonates with pneumonia and negative NP swab.
Kirtsman et al. (Toronto, Canada)[123]	35 w + 5 d, 40 y old, CS, maternal familial neutropenia and gestational diabetes, symptomatic	RT-PCR	1	NP swab positive on day of birth (no skin-to-skin contact), day 2 and day 7. Plasma positive on day 4 and stool positive on day 7; breastfeeding otherwise keeping a distance; breast milk positive.
Knight et al. (UK) [9]	244 cases, late second and third trimester; 4VD, 8CS	RT-PCR	12	12 infants (5%) positive for SARS-CoV-2 RNA, 6 of these infants within the first 12 h after birth.
Mehta et al. (New Jersey, USA) [124]	dichorionic twins, 28 w, 39 y old, IVF, CS, symptomatic	RT-PCR	1	One twin tested positive 72 h after birth.
Patané et al. (Bergamo, Italy; * possible duplication with Ferrazzi et al.) [97]	22 cases, case 1: 37 w + 6 d, VD, symptomatic case 2: 35 w + 1 d, CS, symptomatic	RT-PCR ISH	(2) *	2 neonates with positive NP swab. Case 1: positive at 24 h and on day 7, NP swab with skin-to-skin contact and breastfeeding. Case 2: positive NP swab on day 7, separation. ISH: positive dots for SARS-CoV-2 spike protein mRNA in the STB of both placentas.
Penfield et al. (NY, USA) [99]	11 cases, 26 w + 5 d–41 w + 3 d, 22-40 y old, symptomatic	RT-PCR	0	1 positive placental swab (CS, severe COVID-19) and 2 positive membrane swabs (between the amnion and chorion membrane; CS, critical COVID-19).
Piersigilli et al. (Brussels, Belgium) [125]	26 w + 4 d, PE and HELLP, CS, symptomatic the day after delivery	RT-PCR	1	Positive NP swab on day 7. Possible pp infection.
Schoenmakers et al. [96], (Rotterdam, The Netherlands)	3rd trimester, obesity and gestational diabetes, CS, asymptomatic at presentation	RT-PCR TEM IHC ISH	0	Positive placenta at maternal and fetal side (RT-PCR). SARS-CoV-2 particles were detected in the STB (TEM, IHC, ISH). Fetal distress but negative for SARS-CoV-2.
Sun et al. (China) [116]	3 cases: 30 w + 5 d–37w; 28-30 y old, CS, symptomatic COVID-19	RT-PCR Chest CT scan	1	1 positive NP swab on day 6. 1 CT on day 6 showed findings suggestive of COVID-19.
Vivanti et al. (Paris, France) [100]	35 w + 5 d, 23 y old, CS, symptomatic	RT-PCR	1	Amniotic fluid, placenta, neonatal blood and non-bronchoscopic bronchoalveolar lavage fluid were positive for E and S genes of SARS-CoV-2. NP and rectal swabs positive: 1 h, 3 d and 18 d postnatal; formula feeding; neonatal viremia.
Wang et al. (China) [117]	40 w, 34 y old, CS, low-grade fever	RT-PCR	1	Positive NP swab 36 h pp; cord blood, placenta and breast milk were negative.
Yu et al. (China) [118] *	7 cases, 39 w + 1 d (37–41 w + 2 d), 32 y old (29–34 y), CS, symptomatic	RT-PCR	1	1 neonate was infected with SARS-CoV-2 36 h after birth (throat swab); placenta and cord blood negative.
Zamaniyan et al. (Sari, Iran) [101]	32 w, 22 y old, CS, severe COVID-19 and controlled hypothyroidism	RT-PCR	1	Positive amniotic fluid and repeated neonatal NP swabs on day 1 and 7 pp; isolated without breastfeeding; maternal death.
Zeng et al. (China) [108]	6 cases, 3rd trimester, CS, mild COVID-19	Serology/ CLIA	(2) *	IgG elevated in 5 neonates, IgM in 2. Infants were isolated from mothers. Negative RT-PCR.
Zeng et al. (China) [119]	33 cases, 3 positives: 31 w + 2 d, 40 w, 40 w + 2 d; CS, symptomatic	RT-PCR	3	NP/anal swabs were positive for SARS-CoV-2 on day 2, 4 and negative on day 6 or 7, respectively.
Zhang et al. (China) [120]	4 cases, CS, symptomatic (3 mothers with symptoms before and 1 after delivery)	RT-PCR	4	Infections detected between 30 h, 5 and 17 days of life (NP and anal swabs). Mild symptoms. No mother-child contact/breastfeeding in 3 cases.
**Total**			**35**	

CLIA, chemiluminescent immunoassays; CS, Caesarean section; IHC, immunohistochemistry; CT, computed tomography; IL, interleukin; ISH, in situ hybridization; IVF, in vitro fertilization; NP, nasopharyngeal; pp, postpartum; RT-PCR, real-time polymerase chain reaction; TEM, transmission electron microscopy; STB, syncytiotrophoblast; vCTB, villous cytotrophoblast; VD, vaginal delivery; y, year; ()* not included in total because of possible duplication or solely elevated IgM antibodies.
